# Accelerated water residual removal in MRS: Exploring deep learning versus fitting‐based approaches

**DOI:** 10.1002/mrm.70031

**Published:** 2025-09-02

**Authors:** Federico Turco, Johannes Slotboom, Milena Capiglioni

**Affiliations:** ^1^ Support Center for Advanced Neuroimaging (SCAN), Institute for Diagnostic and Interventional Neuroradiology University of Bern Bern Switzerland; ^2^ Graduate School for Cellular and Biomedical Sciences (GCB) University of Bern Bern Switzerland

**Keywords:** 3D‐MRSI, deep learning frameworks, GPU optimization, torch auto‐differentiation, water residual removal

## Abstract

**Purpose:**

Removing water residual signals from MRS spectra is crucial for accurate metabolite quantification. However, currently available algorithms are computationally intensive and time‐consuming, limiting their clinical applicability. This work aims to propose and validate two novel pipelines for fast water residual removal in MRS.

**Methods:**

Two methods for water residual removal are proposed and evaluated: DeepWatR and WaterFit. DeepWatR uses a U‐Net‐like architecture with an attention mechanism for skip connections. WaterFit uses Torch auto‐differentiation to estimate parameters for a 7‐pool Lorentzian model. The accuracy and time‐efficiency of these methods were assessed using simulated and in vivo 1H brain datasets and compared with the gold standard, Hankel Lanczos singular value decomposition method (HLSVD)Pro.

**Results:**

In the simulated dataset, the average percentage quantification error was (8.54±17.49)% for DeepWatR and (7.86±16.69)% for WaterFit, both comparable to (7.68±15.6)% for HLSVDPro in the main metabolites (Cr, Cho, and NAA). DeepWatR was 51 times faster and WaterFit was 22.7 times faster than HLSVDPro for a dataset of 10 000 voxels when using a low‐end graphics processing unit. For 100 voxels, the speed‐up is 6.5 and 7.5 times faster for DeepWatR and WaterFit, respectively. WaterFit showed higher metabolite fitting accuracy after water removal compared to DeepWatR.

**Conclusion:**

WaterFit showed a superior balance of accuracy and processing speed in removing water residual from MRS data compared to DeepWatR. The proposed WaterFit implementation significantly reduces preprocessing time while maintaining metabolite fitting accuracy comparable to gold standard methods. This advancement addresses the need for efficient processing methods that can facilitate analysis and enhance the clinical utility of MRS.

## 1 INTRODUCTION

MRS aids in diagnosing and stratifying multiple neuropathologies by quantifying the presence of specific metabolites in the brain.^1^, ^2^, ^3^, ^4^ However, because of its abundance and intensity in biological tissues, the strong water signal compromises MRS data quality, making water removal a critical step for accurate metabolite quantification.^5^ Water removal can be partially achieved during signal acquisition using methods such as the chemical shift selective water suppression (CHESS)^6^ or variable pulse power and optimized relaxation delay (VAPOR).^7^ Although these approaches are effective for single voxel spectroscopy, the influence of $B_{0}$ inhomogeneities in 2D/3D echo‐planar spectroscopy imaging (EPSI) decreases water suppression performance,^5^ leaving a water residual signal that must be removed during postprocessing.^5^

There are different approaches to remove water residual in MRS spectra, including the application of a finite impulse response filter, as proposed by Marion et al.^8^ and the subsequently developed alternative.^9^ However, the most recommended approaches are based on singular value decomposition (SVD),^10^ particularly the Hankel Lanczos singular value decomposition method (HLSVD)^11^ and its variant HLSVDPro.^12^ Both SVD‐based methods remove water from MRS spectra by decomposing the signal into exponentially damped sinusoids and removing those signals in a user‐defined frequency range around the main water resonance. HLSVDPro offers superior time performance compared to HLSVD.^13^ However, HLSVDPro still involves significant computational effort, resulting in long processing times, especially for modern MRSI sequences with spatially resolved signals, which greatly increase the number of spectra to be processed. Recent approaches have focused on improving time performance, such as L2 regularization^14^ and Casorati matrix singular value decomposition.^15^ Although these methods obtain a strong acceleration, they are fairly new and need thorough validation.

We propose and evaluate two approaches for accelerated water residual removal to reduce computational time. The first approach, DeepWatR, is based on a UNet deep learning (DL) architecture inspired by previous research that successfully applied UNets to process spectroscopy and spectrum‐like data.^16^, ^17^, ^18^, ^19^, ^20^ Given the simplicity and low computational cost of the required forward operations, we hypothesize that this implementation will provide a significant speed‐up. Despite the expected acceleration, DL methods are also known to be susceptible to bias.^21^ Therefore, we also proposed an alternative method, named WaterFit, implemented as a linear combination model fitting seven Lorentzian shapes. This approach is based on TensorFit, a fast metabolite fitting tool, which achieves a substantial increase in computational speed while maintaining similar accuracy and robustness as standard algorithms.^22^ Both implementations benefit from using graphics processing units (GPUs) to accelerate the processing time. We analyzed the performance of each method in comparison to the gold standard HLSVDPro, focusing on the trade‐off between acceleration and accuracy in both simulated and in vivo data.

## 2 METHODS

### 2.1 DeepWatR implementation

**FIGURE 1:**
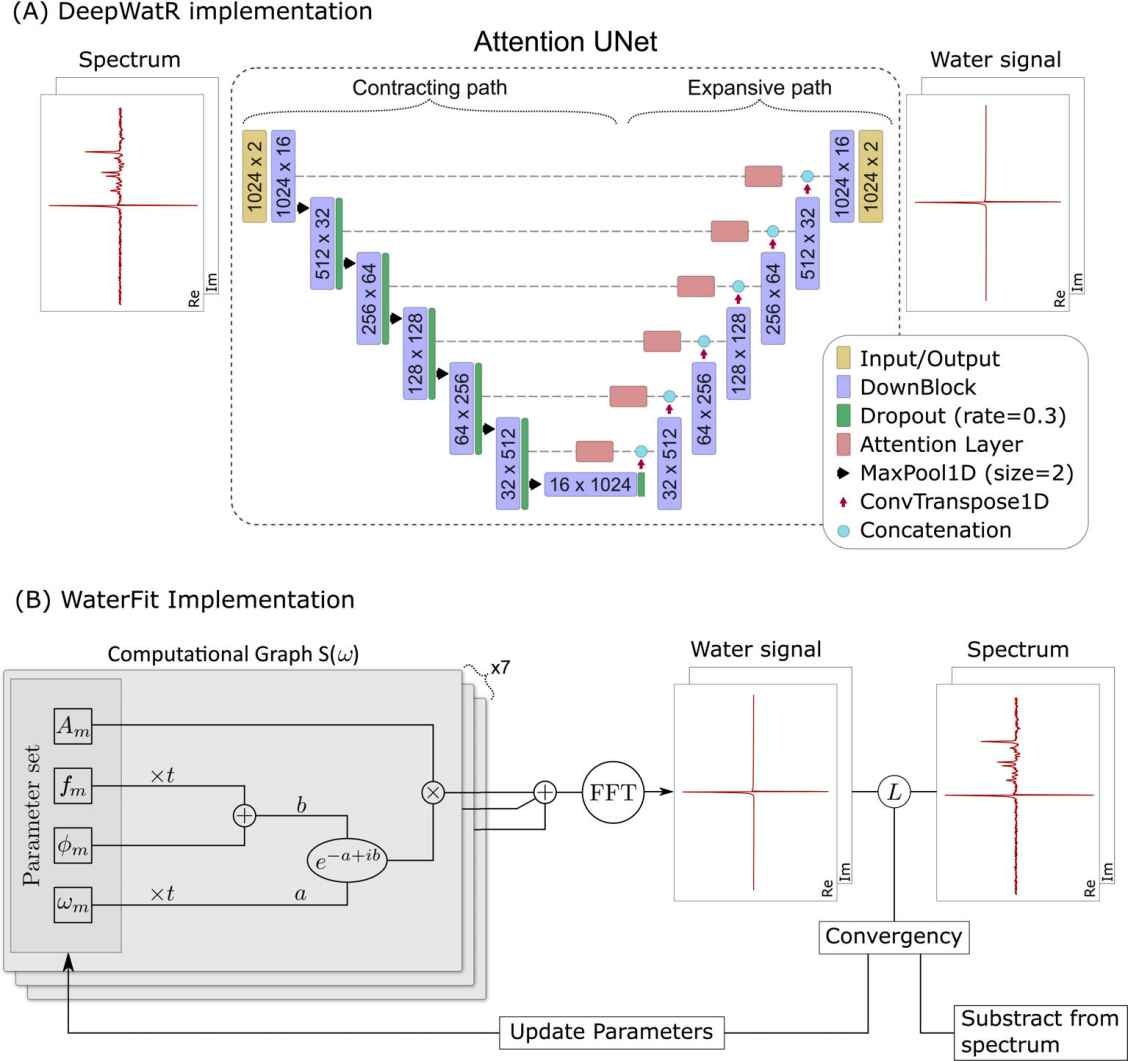
(A) DeepWatR implementation: an attention U‐Net designed to generate water peaks. The input and output dimensions are (batch size, 2, 1024), where the second dimension represents the real and imaginary parts of the spectrum. (B) WaterFit implementation: uses PyTorch's computational graph for fitting water peaks. Both the Water signal (modeled) and Spectrum (target) have the same dimensions as in (A). L denotes the loss function used for error minimization. Before the fast Fourier transform (FFT), the plus sign represents the sum of the seven computed Lorentzian signals.

The DeepWatR implementation uses an Attention‐UNet^23^ neural network to replicate and subtract the water peak from the original spectrum. As illustrated in Figure 1A, the network architecture comprises six convolutional blocks (ConvBlock) as a contracting path. Each ConvBlock consists of the successive application of the following layers: Conv1d, BatchNorm1d, LeakyReLU, Conv1d, BatchNorm1d, and LeakyReLU. The output dimensions of each block are denoted as N×M, where N represents the input length and M indicates the number of features. The first Conv1d layer in each ConvBlock increases the feature size from the input to the output dimensions. To prevent overfitting, dropout layers with a rate of 0.3 are applied to all blocks except the first.

After each ConvBlock, the output is saved for a skip connection and serves as input for the next ConvBlock, with frequency dimensionality reduced by a factor of two using MaxPool1d. Each skip connection feeds into an attention layer to improve the accuracy of the water peak replication while minimizing the contribution of the metabolite peaks to the output. Specifically, the attention mechanism generates a spatially varying “attention map” that highlights the spectral features most relevant to water peak reconstruction while suppressing those linked primarily to metabolite peaks. This guides the network to focus on regions that contain strong water‐signal and minimize the influence of metabolite signals in the final water‐peak reconstruction. This layer is implemented as described in the original attention mechanism publication.^24^

On the expansive path, the first layer is a transpose convolution (ConvTranspose1d), which increases the spatial dimensionality to be concatenated, across the features dimension, with the attention layer from the previous level's skip connection. The resulting tensor is fed into a ConvBlock. This process is repeated until reaching the highest level of the UNet, where the ConvBlock output undergoes an additional set of convolutions to reduce the number of features to two: the real and imaginary water signals. We implemented the described UNet using Torch 2.3.0^25^ with CUDA 12.1 for GPU use.

The input to the UNet is the unfiltered spectrum signal (from now on referred to as the input) in the frequency domain, with the real and imaginary parts represented as two features. The resulting input shape is ($B_{s}$, 2, 1024), where 1024 is the length of the signal, and $B_{s}$ is called batch size and represents the amount of spectra fed to the network at the same time to improve the training procedure. The UNet produces an output of the same dimensions and will reconstruct only the water peak. The training dataset consists of pairs of full spectra and their corresponding water‐only spectra, which serve as the target outputs. The loss is then calculated against the water peak generated by HLSVDPro and used for training. This loss function is defined as:

(1) $$\begin{array}{ll} L\left(W\left(\omega \right),W^{\prime }\left(\omega \right)\right) & =\mathrm{MSE}\left(W\left(\omega_{\left[4.4,-\right]}\right),W^{\prime }\left(\omega_{\left[4.4,-\right]}\right)\right)\\ & \;+\lambda \;\mathrm{MSE}\left(W\left(\omega_{\left[0,4.4\right]}\right),W^{\prime }\left(\omega_{\left[0,4.4\right]}\right)\right), \end{array}$$

where W(ω) is the water signal generated by the network and $W^{\prime }\left(\omega \right)$ is the target water peak. $\omega_{\left[a,b\right]}$ denotes the frequency range between a and b in ppm. In the case of $\omega_{\left[a,-\right]}$, the range is between a and the end of the spectrum. The term MSE represents the mean squared error between W and $W^{\prime }$ in the shown frequency range. The parameter λ represents the weight of the loss evaluated in the water versus the metabolite region. We determined the optimal λ by evaluating the performance on the validation dataset (20% of the total dataset). We found that a value of five improved the robustness of the network in generating the water signal while minimizing artificial artifacts in the metabolite range, therefore, reducing metabolite fitting errors.

During training, validation, and testing, both the input and output spectra were normalized on both the real and imaginary parts, to the maximum value in the whole spectrum. This normalization ensures that large variations in the magnitude of water peaks do not introduce bias. Before subtracting the water peak from the original spectrum, the water peak was scaled back by the normalization factor.

### 2.2 WaterFit implementation

To explore a more deterministic approach with no risk of hallucinations or biases,^21^ we implemented a fitting tool that generates the water residual as a linear combination of seven Lorentzian peaks and fits this model to the input spectra. This approach is based on the TensorFit implementation described in Turco et al.,^22^ where the model is implemented as a computational graph in Torch, and the free parameters of the model correspond to each Lorentzian parameter. Figure 1B shows a schematic of this implementation where the water signal in frequency domain, W(ω), is represented as the sum of these seven Lorentzian peaks:

(2) $$W\left(\omega \right)=\mathrm{FFT}\left(\sum_{m=1}^{7}A_{m}e^{-\omega_{m}t+i\left(2\pi f_{m}t+\phi_{m}\right)}\right),$$

where $A_{m}$, $\omega_{m}$, $f_{m}$, and $\phi_{m}$ represent the Lorentzian amplitude, damping, frequency shift, and phase, respectively. The number of Lorentzian peaks was chosen to contain the maximum number of peaks found by HLSVDPro, which in the training dataset was six. This number can be lowered for a faster fitting in exchange for accuracy in the water removal.

The fitting process begins by evaluating the computational graph to generate a modeled water signal, for which a loss function is computed between the water peak and the target spectra. The gradient of the loss with respect to each parameter is used to update them toward minimizing the loss function. By iteratively applying this step we minimize the loss and extract the optimal set of parameters to represent the water peak. An advantage of this approach is that it leverages the GPU acceleration, enabling highly parallelized fitting across hundreds of spectra simultaneously. The loss function used for minimization is defined as follows:

(3) $$\begin{array}{ll} L\left(W\left(\omega \right),S\left(\omega \right)\right) & =\mathrm{MSE}\left(W\left(\omega_{\left[4.4,7.0\right]}\right),S\left(\omega_{\left[4.4,7.0\right]}\right)\right)\\ & \;+\lambda \left\|W\left(\omega_{\left[0.4,4.4\right]}\right)\right\|+\sum_{m=1}^{7}A_{m}^{2}, \end{array}$$

where S(ω) is the full spectrum to which the water is being fitted. The term $\left\|W\left(\omega_{\left[0.4,4.4\right]}\right)\right\|$ penalizes the area of the Lorentzian tail that overlaps with the metabolite region. We selected the λ value that yielded the optimal performance for each method on the validation dataset, in this case, the optimal value was one. The last term of the loss function penalizes peaks in proportion to the square of their amplitude. This prevents abnormally large peaks in the water region from improving the fit while introducing distortions to the baseline in the metabolite region.

To prevent Lorentzian components from leaking into the metabolites region, the allowed frequency shift range for each peak was constrained between 4.4, and 5.0 ppm using the *torch.clamp* function. Finally, the loss was minimized using the Rprop optimizer^26^ with a learning rate of 1, and early stopping after 20 iterations if the error improvement was less than 0.1%.

The initial values for WaterFit were 0 a.u., 30 Hz, and 0° for the amplitude, damping, and phase of each Lorentzian, respectively. For the frequency shift, the initial seed was uniformly distributed in the range of the water peak, this is: f= [4.7, 4.75, 4.65, 4.80, 4.60, 4.85, 4.55] ppm.

### 2.3 Simulated dataset

To evaluate the accuracy of metabolite quantification with each method, we simulated a dataset including nine metabolites: Cho, glutamate (Glu), lactate (Lac), glutamine (Gln), Cr, aspartate (Asp), N‐acetyl aspartate ^2^CH_3_ chemical group (NAA), myo‐inositol (mI), and another N‐acetyl aspartate ^3^CH_2_ chemical group. This dataset was simulated following the procedure and parameter ranges as previously detailed in Turco et al.,^22^ with a frequency shift, damping factor and phase chosen from an uniform distribution between {−20, 20} Hz, {1.5, 6} Hz, and {−π/4, −π/4}°, respectively. The amplitude varied depending on the metabolite, by multiplying the model initial values by a scaling factor chosen randomly between {0.1, 2} around the base values shown in Table 1. For realism, we added a Gaussian noise with a SD that was set to have a SNR randomly sampled between 5 and 50.

**TABLE 1:** Base values for each metabolite amplitude used in the simulated data, from which amplitude values were randomly generated.

	NAA	Cho	Cr	Gln	NAA_[2.6ppm]_	Lac	mI	Glu	Asp
Base value [a.u.]	1.12	8.83	1.33	1.12	5.46	0.83	1.71	0.43	5.93

Abbreviations: Asp, aspartate; Gln, glutamine; Glu, glutamate; Lac, lactate; mI, myo‐inositol.

To simulate the water residual, a set of 5 Lorentzian peaks were considered as proposed by Lin et al.,^14^ computed as:

(4) $$W\left(\omega \right)=\mathrm{FFT}\left(\sum_{k=1}^{5}A_{k}e^{-w_{k}t+i\left(2\pi f_{k}t+\phi_{k}\right)}\right),$$

where $A_{k}$, $w_{k}$, $f_{k}$, and $\phi_{k}$ are the Lorentzian amplitude, damping, frequency shift, and phase, respectively. Each parameter was randomly chosen around the initial values in Table 2. The amplitude of each peak k ($A_{k}$) was sampled from a uniform distribution around the initial value $A_{k,0}$ as $A_{k}=A_{k,0}U\left(10,150\right)$, where U(a,b) corresponds to a uniform distribution between a and b. The damping factor is varied as $w_{k}=w_{0,k}U\left(0.3,1.7\right)$. The frequency shift and phase are selected as $f_{k}=f_{0,k}+0.1N\left(0,1\right)$ ppm and $\phi_{k}=N\left(0,\pi \right)$, where N(a,b) is a normal distribution with mean a and SD b. The distribution of values was based in Lin et al.,^14^ in combination with statistical analysis of the peaks removed with HLSVDPro on the dataset described below.

**TABLE 2:** Initial parameter values for water simulation, including amplitudes, damping factors, frequency shifts, and phases used as baseline values for parameter variations for each peak.

Lorentzian peak no.	Amplitude $A_{0}$ [a.u.]	Damping $w_{0}$ [Hz]	Frequency shift $f_{0}$ [ppm]	Phase $\phi_{0}$ [°]
1	1	40	4.7	0
2	0.33	40	4.78	0
3	0.33	40	4.62	0
4	0.11	40	4.85	0
5	0.11	40	4.55	0

Abbreviations: $A_{0}$, amplitudes; $w_{0},$ damping factors; $f_{0}$, frequency shifts; $\phi_{0}$, phases.

### 2.4 In vivo dataset

In addition to simulated data, we used three different types of in vivo data: a training dataset for DeepWatR, a testing dataset for both DeepWatR and WaterFit, and an extra dataset containing diverse water peaks to assess robustness. All the cases were acquired with a semi‐LASER 2D‐MRSI sequence with acquisition parameters: acquisition bandwidth = 1199 Hz, number of time points = 1024, and TR = 1500 ms.

The first dataset, used for training and validating DeepWatR comprised a total of 2688 spectra from 12 subjects. This included 1344 spectra from six subjects scanned at 1.5 T, with 672 spectra acquired at TE = 40 ms and 672 at TE = 135 ms. The remaining 1344 spectra were from another six subjects scanned at 3 T, with 672 spectra at TE = 40 ms and 672 at TE = 135 ms. We used HLSVDPro with a 512 × 512 Hankel matrix and 30 singular values in the decomposition. From the 30 Lorentzians generated, we selected those with frequency shift was between 4.4 and 5.0 ppm and sum them to obtain the water peak in each input case. The total response of the water peak was saved to be used as a label (output) in DeepWatR. To increase the amount of data we can use for training and validating the network, we augmented the data to have higher variability and diversity in our dataset. For this, we removed the water from the input spectrum to obtain a clean spectrum and created a set of 2668 spectra without water (S(ω)), and its corresponding water peak (W(ω)). To augment the amount of training data, to each spectrum $S_{i}\left(\omega \right)$ was added to a randomly modified water peak $W_{j}\left(\omega \right)$ (i≠j) randomly selected, this is $S_{\mathrm{new}}\left(\omega \right)=S_{i}\left(\omega \right)+T\left(W_{j}\left(\omega \right)\right)$. The transformation T corresponds to modifying the frequency shift, a phase, damping, and amplitude to the original water peak. This is:

(5) $$T\left(W\left(\omega \right)\right)=\mathrm{FFT}\left(A^{\prime }e^{-w^{\prime }t+i\left(2\pi f^{\prime }t+\phi^{\prime }\right)}{\mathrm{FFT}}^{-1}\left(W\left(\omega \right)\right)\right),$$

where $A^{\prime }$, $w^{\prime }$, $f^{\prime }$, and $\phi^{\prime }$ correspond to a randomly selected area, damping, frequency shift, and phase, respectively. $w^{\prime }$ is sampled from a uniform distribution U(−4, 10 Hz), $f^{\prime }$ from U(10, 10 Hz), $\phi^{\prime }$ from U(−π,π), and $A^{\prime }$ is applied to have the same maximum amplitude before and after the transformation. This transformation was done four times, multiplying the available data by five, to a total of 13 440 spectra. The dataset was split into 80% and 20% for training and validation, respectively.

The second dataset, used for testing performance of both DeepWatR and WaterFit against HLSVDPro, consisted of a total of 3360 spectra acquired on 13 patients with healthy tissue scanned at 3 T. The acquisition used the same sequence and echo times (1568 spectra for TE = 135 ms and 1792 for TE = 40 ms) as the training dataset. For HLSVDPro the configuration is the same as before.

The third dataset used for testing comprises clinical data from a patient with a tumor. It includes pre‐ and post‐surgery MRSI scans conducted on a 1.5 T scanner with the same semi‐LASER sequence. This dataset contains 256 spectra from each state of the follow‐up, with the post‐ spectrum exhibiting a broad and large water peak. In this case, the water range in HLSVDPro and WaterFit was increased to the range 4.4 to 5.4 ppm.

The end goal of MRS analysis is the accurate quantification of metabolites. Therefore, we performed the metabolite fitting step on the simulated data to assess accuracy, and the in vivo data to compare with HLSVDPro. The quantification was performed using TensorFit^22^ with a spectral model formed by the nine metabolites used in the dataset simulation section above. The model includes a baseline represented with a linear combination of cubic splines, resulting on a spectral response given by:

(6) $$S\left(\omega \right)=\mathrm{FFT}\;\left(\;e^{-T_{2,c}^{-1}\;t+i\left(2\pi \omega_{c}t+\phi_{c}\right)}\sum_{m=1}^{N}A_{m}B_{m}\left(t\right)\right)+\alpha \;S_{b}\left(\omega \right),$$

were $A_{m}$, $\phi_{c}$, $\omega_{c}$, $T_{2,c},$ are the m‐th metabolite amplitude, and commons zero‐order phase, frequency shift, and damping factor, respectively. Additionally, $S_{b}\left(\omega \right)$ represents a matrix where each row is a cubic spline, composed of 50 splines with centers uniformly distributed across the whole frequency range. The amplitude of each spline is represented by $\alpha =\left(\alpha_{0},\alpha_{1},\ldots ,\alpha_{49}\right)$ were the component $\alpha_{i}$ corresponds to the amplitude of the i‐th spline. This baseline accounts for macromolecular contributions as well as for remanent water influence. The metabolite fitting using TensorFit was performed with the exact same configuration, independently of the method used to remove water. The percentage error for each amplitude parameter on the simulated dataset was computed as: $\text{error}\left[\%\right]=100*\left(A\prime_{m}-A_{m}\right)/A_{m}^{\prime }$, where $A\prime_{m}$ and $A_{m}$ represent the groundtruth and the fitted amplitude, respectively.

### 2.5 Time efficiency comparison

To estimate the execution time of each method, we used a simulated dataset, which provided a large volume of data. For DeepWatR and WaterFit, we used an NVIDIA GTX 1060 and 2080ti GPU with 6 and 11 GB of memory. For the HLSVDPro implementation, we parallelized the process across 16 processes using an AMD Ryzen 7 2700X CPU. The reported times reflect only the water removal step, excluding data loading and preprocessing. When evaluating DeepWatR, the removal process was performed in batches of 512 spectra because of memory limitations of the used GPU, this could be is modified to higher numbers, but this value guarantees that the whole process fits within any modern GPU's memory (NVIDIA GTX 1060 or newer).

## 3 RESULTS

### 3.1 Analysis on simulated data

**FIGURE 2:**
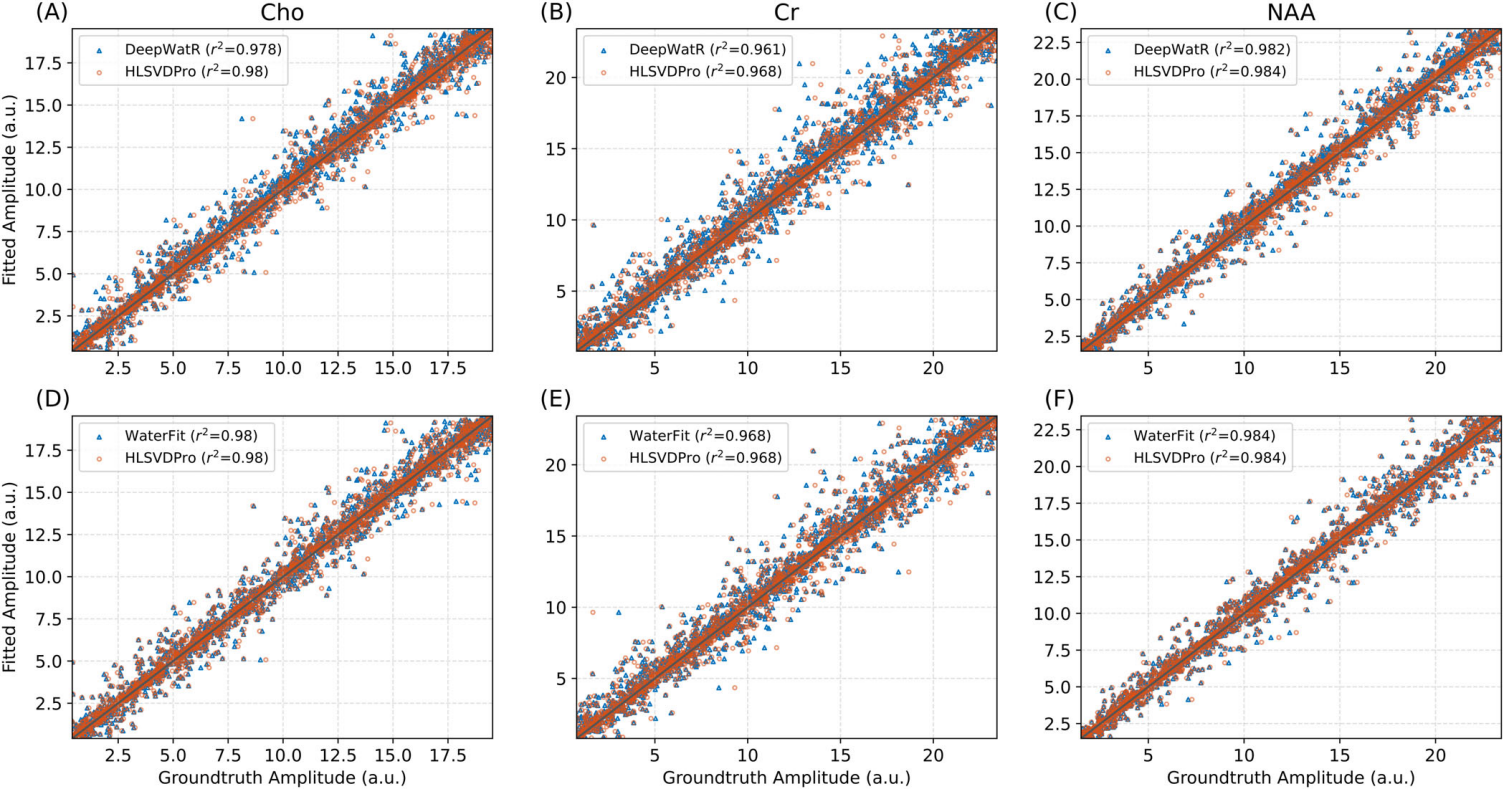
Scatter plots comparing ground truth metabolite amplitudes to those fitted using our implementations and HLSVDPro, based on simulated spectra. The first and second rows correspond to DeepWatR and WaterFit, respectively. The columns correspond to the three main metabolites Cho, Cr, and NAA, from left to right.

Figure 2 presents a comparative scatter plot analysis of metabolite fitting results from DeepWatR, WaterFit, and HLSVDPro, evaluated against ground truth values in the simulated dataset. Figure 2A–C shows that although DeepWatR performs similarly to HLSVDPro, it exhibits slightly higher variance and a lower R^2^ value with a statistically significant difference in a t test comparison with HLSVDPro with (p > 0.01). In contrast, Figure 2D–F demonstrate that WaterFit produces results almost identical to those of HLSVDPro, with R^2^ differences on the order of 10^−4^ and no statistically significant difference (p > 0.01). The statistical significance was calculated with a Student's t‐test between the metabolite's amplitudes distribution obtained after each method. A *p*‐value smaller than 0.01 was considered statistically significant.

Table 3 shows the average percentage error across the entire dataset for the metabolite fitting step for each metabolite and method. For all metabolites, except Lac and mI, WaterFit demonstrates a smaller percentage error than DeepWatR, with statistically significant differences (*p* < 0.01) in all metabolites except for NAA_[2.6ppm]_, Asp, and Glu.

**TABLE 3:** Percentage error between the metabolites fitting on the dataset obtained by water removal using each method and the ground truth used in the simulation.

Method	NAA	Cho	Cr	Gln	NAA_[2.6ppm]_	Lac	mI	Glu	Asp	Average
HLSVDPro	5.41	8.66	8.98	21.84	45.19	54.55	54.16	97.86	129.67	47.37
WaterFit	5.42	8.82	9.33	21.78	45.37	61.74	56.8	97.82	135.86	49.22
DeepWatR	5.95	9.28	10.4	22.56	46.85	57.59	56.73	98.44	138.67	49.61

Abbreviations: Asp, aspartate; Gln, glutamine; Glu, glutamate; Lac, lactate; mI, myo‐inositol.

### 3.2 Analysis of in vivo data

**FIGURE 3:**
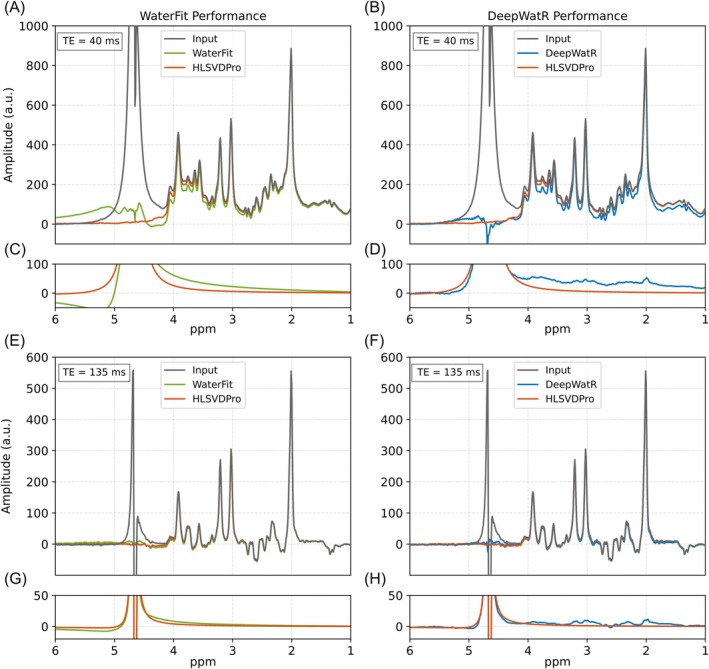
Water removal performance in vivo. Average spectra with and without water residual for the whole in vivo dataset, compared against HLSVDPro and the original spectra. The first (A‐B) and third (E‐F) rows correspond to TE 40 and 135 ms, whereas the first and second columns correspond to WaterFit and DeepWatR implementations, respectively. Additionally, (C) and (D) show a closer view of the water peak in the metabolite range found by WaterFit and DeepWatR for TE = 40 ms, respectively. Subplots (G) and (H) show the water peak in the case of TE = 135 ms.

Once the performance was established for simulated datasets, we tested both implementations on in vivo data. Figure 3 shows the average spectra in frequency domain over the whole dataset. Figure 3A,B shows water removal performance for the short TE dataset using WaterFit and DeepWatR, respectively. DeepWatR exhibits a small but constant frequency domain offset and hallucinated artifacts in the metabolite region as shown in Figure 3D in the final spectra compared to HLSVDPro, whereas the difference between WaterFit and HLSVDPro decays with the spectral distance to the water resonance as shown in Figure 3C, consistent with the Lorentzian shape. Figure 3E,F presents the same comparison for the long echo time (TE = 135 ms), where the narrower water peak is effectively removed by both methods, resulting in no residual baseline. Although HLSVDPro performance is better in the absorption (real) component of the spectrum, its remanent water in the dispersion (imaginary) component is bigger as shown in Figure S1. For a better comparison on the performance in the water range, Figure S2 shows the area distribution under the water‐free remanent signal in both the real and imaginary parts.

**FIGURE 4:**
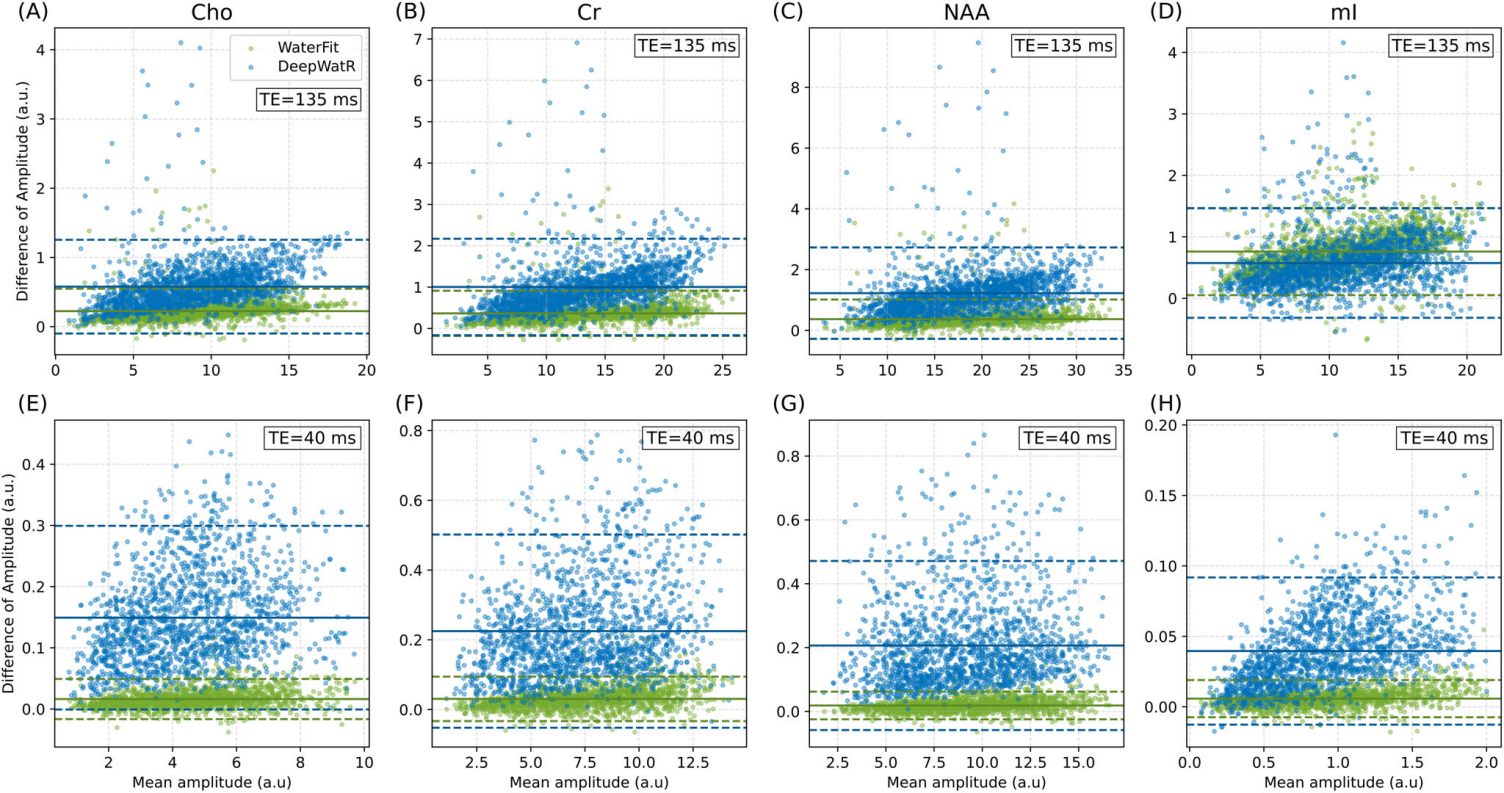
Metabolite fitting in vivo performance. Bland‐Altman plot between the main metabolites amplitudes removing water with DeepWatR and WaterFit, against the result obtained with the gold standard HLSVDPro for in vivo data. (A–D) Correspond to TE = 135 ms and (E–H) to TE = 40 ms. The solid horizontal line corresponds to the bias (average of all points) and the dashed lines represent ±1.96 SDs, in blue and green for DeepWatR and WaterFit, respectively.

Given the lack of ground truth for the in vivo dataset, we compare both methods against HLSVDPro, which is considered the gold standard and performed best on the simulated data. To evaluate this for both WaterFit and DeepWatR, we present the Bland‐Altman plot of the fitting results for the main metabolites (Cho, Cr, and NAA) and mI. The plot shows the distribution of the difference between the metabolite amplitude obtained by HLSVDPro and the amplitude obtained by our method in the y‐axis as a function of the mean between both values. The solid line corresponds to the average of the distribution and represents the bias, whereas the dashed line shows the interval of 1.96 SDs from the mean. For the dataset with TE = 40 ms. Figure 4A–D shows the result for Cho, Cr, NAA, and mI, respectively, The green scatter is the fitted amplitude for WaterFit, whereas blue corresponds to DeepWatR, and both are compared against HLSVDPro. Although both methods exhibit a clear bias, WaterFit shows a lower bias compared to DeepWatR, except in the case of mI, where WaterFit bias is slightly bigger. DeepWatR demonstrates lower correlation, primarily because of artificial artifacts introduced by the network in the metabolite region as shown in Figure S3. WaterFit does not introduce such artifacts because the decay is purely Lorentzian within the 4.4 to 0 ppm range, which makes the water peak leaking into the metabolite region smooth enough to be correctly fitted by the baseline.

Figure 4E–H displays the result for TE = 135 ms for the same metabolites. In this case, both methods show high correlation with HLSVDPro, but the scatter shows a slight bias to smaller metabolite amplitudes for DeepWatR. This corresponds to a remaining baseline on the metabolite region, not correctly modeled by the baseline of the metabolite fitting model.

In most cases, there is an overestimation on the water peak amplitude in the metabolite region for both WaterFit and DeepWatR. This effect can be seen in Figure S4 that presents the mean difference between HLSVDPro and our methods in the metabolite region as a function of the water concentration, where we can see that in the majority of the cases the water residual overlapping with the metabolite region is smaller in HLSVDPro. Additionally, to better understand the influence of the water amplitude in the final spectrum, Figure S5 shows example of each method performance in low‐ and high‐water concentrations for both echo times.

**FIGURE 5:**
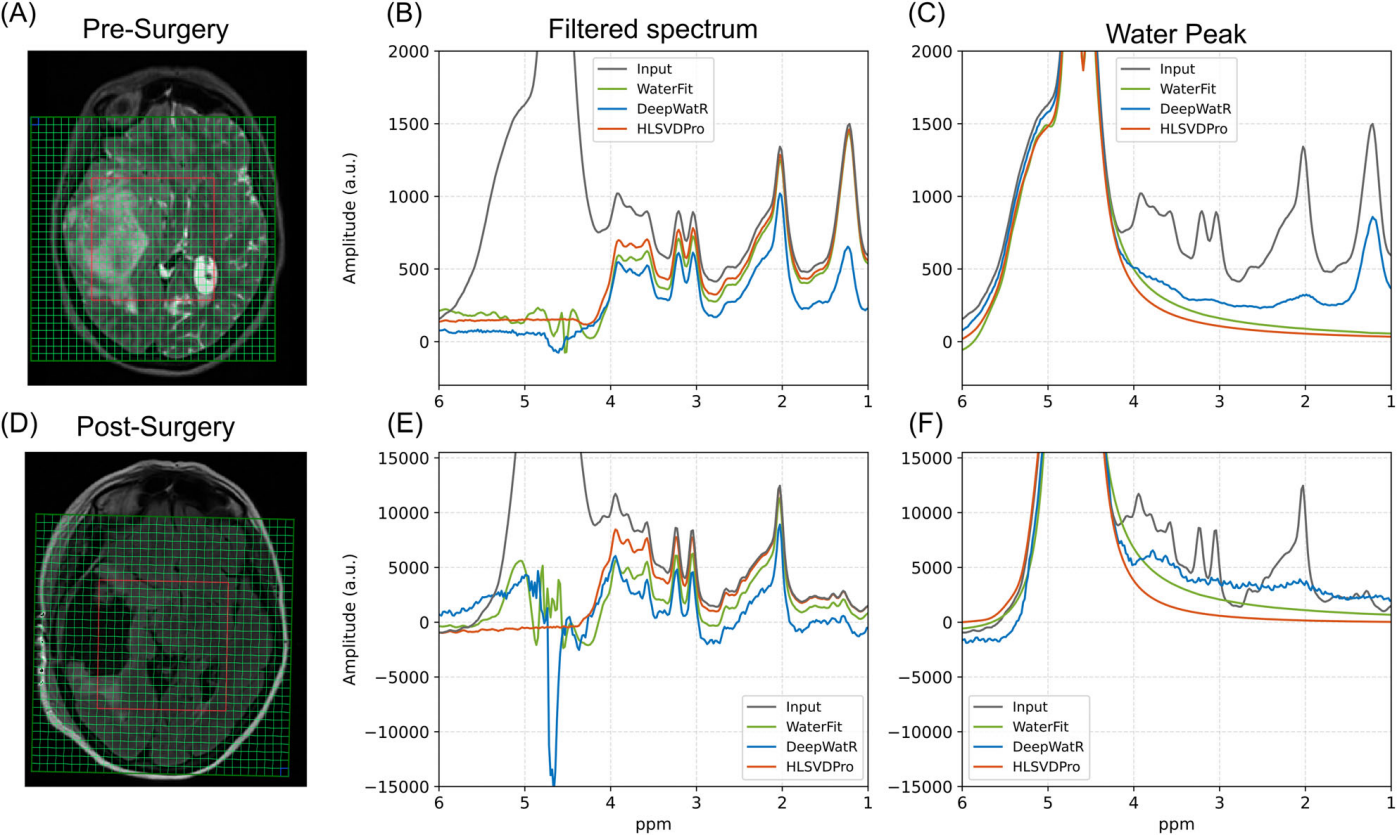
Water removal performance on a clinical case of tumorous tissue for each method. (A) Shows the acquired grid and (B) and (C) show the average across all voxels of the filtered spectrum and water peak, respectively. Presenting the results for DeepWatR (blue), HLSVDPro (orange), WaterFit (green), and the corresponding initial spectrum (dark gray). (D) Shows the acquired grid after tumor removal, (E) and (F) correspond to the average across all voxels for the filtered spectrum and the extracted water peak, respectively, and in this case with bigger and broader water residuals.

To evaluate performance on broader and larger water peaks, we tested both methods in a clinical dataset of a tumor patient. Figure 5A–C depicts the averaged results in a pre‐surgery scan. In the case of Figure 5B, it shows the average signal across all voxels after removing water with all three methods, and Figure 5C presents only the averaged water peak. The DeepWatR implementation exhibits superior performance in removing residuals in the water range. However, a small hallucination is seen in the metabolite range. Instead, the decaying nature of both HLSVDPro and WaterFit forbids any artificially added artifacts in the metabolite region.

In the post‐surgery case shown in Figure 5D, it can be noticed in the average signal across all the voxels that DeepWatR introduces a distinct artificial peak in the water range (Figure 5E) and a noticeable baseline artifact in the lower ppm range (Figure 5F). In contrast, WaterFit shows some residual signal in the water range, but no major influence in the metabolite fitting compared to HLSVDPro.

### 3.3 Time efficiency

**FIGURE 6:**
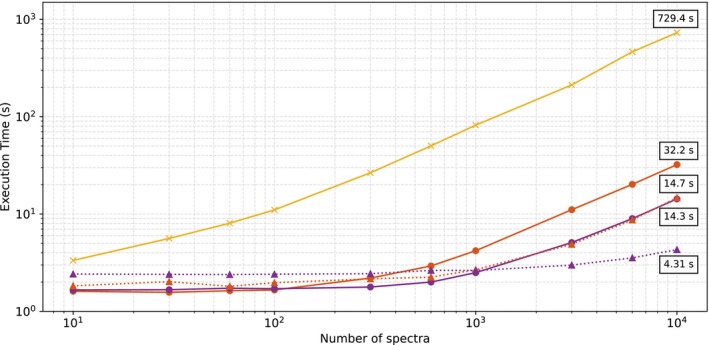
Execution time of DeepWatR, WaterFit, and the gold standard HLSVDPro as a function of the number of spectra in log–log scale.

To evaluate the efficiency of various implementations for water removal from large datasets, we measured the time required to process complete datasets. Figure 6 shows the performance of each method as a function of dataset size, ranging from 10 to 10 000 spectra for two GPU, an NVIDIA GTX 1060 with 6 GB of memory, and a NVIDIA GTX 2080ti with 11 GB. The execution time of HLSVDPro increases consistently with dataset size because of its limited parallelization, and operating on only 16 processes. In contrast, WaterFit and DeepWatR demonstrate stable performance up to 100 and 300 spectra, respectively, thanks to GPU acceleration. For the largest dataset of 10 000 spectra, we found WaterFit and DeepWatR to be 22.7 and 51 times faster than HLSVDPro, respectively. For the GTX 1060, this speed‐up corresponds to an execution time of 729, 32, and 14 s for HLSVDPro, WaterFit, and DeepWatR, respectively. Moreover, a more specialized GPU as the GTX 2080ti yields a faster execution time, of approximately 170 and 50 times faster than HLSVDPro for DeepWatR and WaterFit, respectively. It must be noted that HLSVDPro cannot take advantage of the GPU because of limitation of the GPU implementations for the SVD.

## 4 DISCUSSION

To enhance the time efficiency of the water removal step in MRS data processing, especially for large datasets, our study implemented and evaluated two advanced methods: DeepWatR, a DL approach using an attention U‐net model, and WaterFit, a fitting method based on a seven‐Lorentzian peak model. Both methods leverage the PyTorch library for GPU acceleration, significantly enhancing processing speed.

When evaluating the performance of the two approaches on simulated data, both DeepWatR and WaterFit exhibited a high correlation with HLSVDPro in terms of metabolite fitting accuracy, with $r^{2}$ values exceeding 0.96. WaterFit demonstrated a slightly higher $r^{2}$ value, indicating a closer match to the ground truth values (Figure 2). On average, WaterFit's metabolite estimations differ from those of HLSVDPro by 0.17%, whereas DeepWatR shows a 0.86% percentage error for the main metabolites (NAA, Cr, and Cho). For the entire metabolite set, the percentage errors are 1.85% and 2.24% for WaterFit and DeepWatR, respectively. The increased error in DeepWatR is attributed to the baseline generated incorrectly in the region of the metabolite, with this effect being more pronounced at shorter echo times, as detailed in Figure S3. Additionally bigger water peaks, with more off‐resonance influence, induce a bigger spurious signal in the metabolite region as shown in Figure 5C,F. These findings suggest that WaterFit may be more reliable for clinical settings requiring precise metabolite quantification, particularly at shorter echo times.

After validating performance with simulated data, we proceeded to test the methods on in vivo acquisitions. Because of the absence of ground truth, we compared the metabolite fitting results of DeepWatR and WaterFit against HLSVDPro, which demonstrated the best performance in simulated data. We observed (Figure 4) that DeepWatR exhibited a noticeable bias at short echo times (TE = 40 ms), attributed to artificial artifacts introduced by the network, which overlap with the metabolite peaks (see Figure S3). In contrast, at longer echo times (TE = 135 ms), DeepWatR demonstrated a reduced bias, with a $r^{2}$ value exceeding 0.99 as shown in Figure S6. WaterFit displayed a smaller overall bias, because of the smooth, decaying nature of the Lorentzian peak model, which better accommodates the characteristics of the data. We observed in Figure 4, that mI, for its proximity to the water peak, was affected more than the rest, where the decaying nature of WaterFit made the over‐estimation less relevant. The baseline fitting of TensorFit effectively accounted for the additional baseline contributions during the quantification step. Therefore, WaterFit is a more reliable method because it minimizes the risk of artificially induced inaccuracies. We attribute the bias toward smaller values in both our implementations to the fact that they generate a water residual in the metabolite region that is, in most cases, slightly bigger than the residual of HLSVDPro, as shown in Figure S4.

To further assess the robustness of the proposed methods against larger and broader water residuals, we tested both implementations on clinical brain tumor data. We found that both WaterFit and HLSVDPro performed well in the tumor region, whereas the DeepWatR approach introduced significant artificial differences in the metabolite range (Figure 5). Despite the presence of noticeable water residuals after applying WaterFit, these should not adversely affect the metabolite fitting process in the tested cases. Both methods introduced a baseline relative to HLSVDPro. However, WaterFit forces residual water to follow a smooth, decaying pattern, which can be modeled during metabolite quantification using a linear combination of third‐degree splines. In contrast, DeepWatR produces a less smooth baseline, leading to biases in metabolite quantification (Figure 4) because of artificial peaks influenced by metabolite signals and the macromolecule baseline (Figure 3D,H). As seen in Figure 4 for mI, this difference in metabolite quantification accuracy decreases for metabolites close to the water range. This indicates that WaterFit may offer better consistency and reliability in clinical environments.

In terms of execution time, WaterFit and DeepWatR demonstrate substantial speed advantages over HLSVDPro when using a GPU, with speed‐ups of up to 22 and 51 times, respectively. For large datasets, such as those with 10 000 spectra, WaterFit and DeepWatR complete the water removal process in 32 s and 14 s, respectively. This makes both methods particularly suitable for high‐resolution spectroscopy where processing speed is a priority.

A key limitation of these methods is their performance in case a suitable GPU is not available. In this scenario, WaterFit takes approximately 510 s, whereas DeepWatR requires approximately 140 s to process the same dataset. This results in speed‐ups of only 1.4 and 5.2 times for WaterFit and DeepWatR, respectively, compared to HLSVDPro. Further limitations of DeepWatR are that the network must be retrained for other acquisition sequences not included in the training dataset or for different number of points in frequency domain, where the current implementation is trained only for 1024 points.

A limitation of this study is that the performance of the proposed methods is only compared with HLSVDPro. Further comparison with newer and faster methods such as L2^14^ regularization and Casorati matrix SVD,^15^ which has shown a strong speed‐up is needed. Additionally, the testing and validation was performed on the same type of data, acquired with the same sequence, echo times, and number of points. A more diverse dataset should be used for more extensive validation. Finally, this study does not consider the cases where the metabolites of interest are down‐field, where the water residual performs worse than the up‐field, and further development should take this into account.

Finally, in future work, WaterFit could model the Lorentzian sum directly in frequency domain, avoiding expensive complex exponentials and Fourier transformations for further time efficiency. Additionally, studying different cost functions and improving the fitting model with additional Gaussian or Voight lineshapes could potentially avoid the induced baseline in the metabolite range. Furthermore, the DL approach could be improved by imposing constraints by using physical models in the network to obtain the Lorentzian parameters as proposed by Gurbani et al.^27^ Although this approach should not lead to an improvement in respect to the WaterFit implementation, because the model auto‐differentiation and the minimization algorithms used during training are essentially the same, it could substantially speed‐up the Lorentzian parameter estimation. A postprocessing alternative, consisting of fitting a single Lorentzian to the water residual in the metabolite range, has the potential to smooth out the signal generated by DeepWatR. This should have a similar performance as WaterFit, for having a water residual easily modeled by a baseline.

## 5 CONCLUSIONS

We propose two methods for water residual removal, the first based on DL and the second on a fitting approach to the water signal. We found that although both methods have good performance, DeepWatR is not robust toward the presence of strong baselines, causing artifacts overlapping the metabolite regions. Instead, WaterFit induces a smooth baseline reassembling the Lorentzian peak tail, which gets correctly modeled by the baseline during the metabolite fitting. With this approach, we obtained a speed‐up in the order of 51 and 22 for DeepWatR and WaterFit, respectively. Therefore, WaterFit significantly reduces the processing time while maintaining acceptable metabolite fitting accuracy, with a potential impact on boosting the clinical use of high‐resolution MRS.

## Supporting information


**Figure S1.** Water removal performance in dispersion mode. Average spectra with and without water residual compared against HLSVDPro and the original spectra. The first and second rows correspond to TE = 40 and TE = 135 ms, while the right and left columns correspond to WaterFit and DeepWatR, respectively.
**Figure S2.** Histogram of the mean amplitude of residual water for both real (top row) and imaginary (bottom row) parts after removal for each method used with TE = 135 (right column) and TE = 40 ms (left columns). The mean and standard deviations of each distribution is shown in each plot legend.
**Figure S3.** Performance of each residual water removal tool, Input corresponds to the original spectrum for a case with TE = 40 ms.
**Figure S4.** Comparison of water suppression performance between WaterFit and DeepWatR using HLSVDPro as a reference in the metabolite region. (A) Mean difference between WaterFit and HLSVDPro (y‐axis) as a functionof the maximum water peak amplitude, for TE = 40 ms (orange) and TE = 135 ms (blue). (B) Same as (A), but for DeepWatR instead of WaterFit. (C) The mean difference between WaterFit and HLSVDPro is plotted against the mean water amplitude, providing an estimate of the peak area. (D) Same as (C), but for DeepWatR.
**Figure S5.** Example spectra showing water residual removal performance for cases with small (left) and large (right) water peaks. Top row (A, B) corresponds to TE = 135 ms; bottom row (C, D) to TE = 40 ms. Original spectra (black) are shown alongside results from DeepWatR (blue), WaterFit (green), and HLSVDPro (orange). Cases in (A) and (C) are from the bottom 10% of water peak area; (B) and (D) from the top 10%.
**Figure S6.** Scatter plot comparing the amplitude of main metabolites (Cho, Cr, NAA) between WaterFit and DeepWatR in the y‐axis, and HSLVDPro in the x‐axis for TE = 40 ms and TE = 135 ms in the first and second row, respectively.

## Data Availability

The model and source code of the algorithms that support the findings of this study will be openly available at https://github.com/TuchoTurco/WaterFit and https://github.com/TuchoTurco/DeepWatR on publication. The in vivo data used for this study can be obtained on reasonable request to the corresponding author.
