# Supplementary material for: Accelerated water residual removal in MRS: Exploring deep learning versus fitting‐based approaches
Source: Magn Reson Med. 2025 Sep 2;95(1):38–50. doi: 10.1002/mrm.70031 (PMC12620168; doi:10.1002/mrm.70031)
Supplement: Supplementary file 1 — Figure S1. Water removal performance in dispersion mode. Average spectra with and without water residual compared against HLSVDPro and the original spectra. The first and second rows correspond to TE = 40 and TE = 135 ms, while the right and left columns correspond to WaterFit and DeepWatR, respectively. Figure S2. Histogram of the mean amplitude of residual water for both real (top row) and imaginary (bottom row) parts after removal for each method used with TE = 135 (right column) and TE = 40 ms (left columns). The mean and standard deviations of each distribution is shown in each plot legend. Figure S3. Performance of each residual water removal tool, Input corresponds to the original spectrum for a case with TE = 40 ms. Figure S4. Comparison of water suppression performance between WaterFit and DeepWatR using HLSVDPro as a reference in the metabolite region. (A) Mean difference between WaterFit and HLSVDPro (y‐axis) as a functionof the maximum water peak amplitude, for TE = 40 ms (orange) and TE = 135 ms (blue). (B) Same as (A), but for DeepWatR instead of WaterFit. (C) The mean difference between WaterFit and HLSVDPro is plotted against the mean water amplitude, providing an estimate of the peak area. (D) Same as (C), but for DeepWatR. Figure S5. Example spectra showing water residual removal performance for cases with small (left) and large (right) water peaks. Top row (A, B) corresponds to TE = 135 ms; bottom row (C, D) to TE = 40 ms. Original spectra (black) are shown alongside results from DeepWatR (blue), WaterFit (green), and HLSVDPro (orange). Cases in (A) and (C) are from the bottom 10% of water peak area; (B) and (D) from the top 10%. Figure S6. Scatter plot comparing the amplitude of main metabolites (Cho, Cr, NAA) between WaterFit and DeepWatR in the y‐axis, and HSLVDPro in the x‐axis for TE = 40 ms and TE = 135 ms in the first and second row, respectively. [file MRM-95-38-s001.pdf]

Supporting Information

Supporting Information 1

Figure S1 shows the average result of water removal for the three methods used, but in the dispersion mode (the imaginary component of the complex signal), as a complement to Figure 3. Subplots A) and B) correspond to TE = 40 ms, for WaterFit and DeepWatR, respectively. C) and D) show the case of long echo time TE = 135 ms.

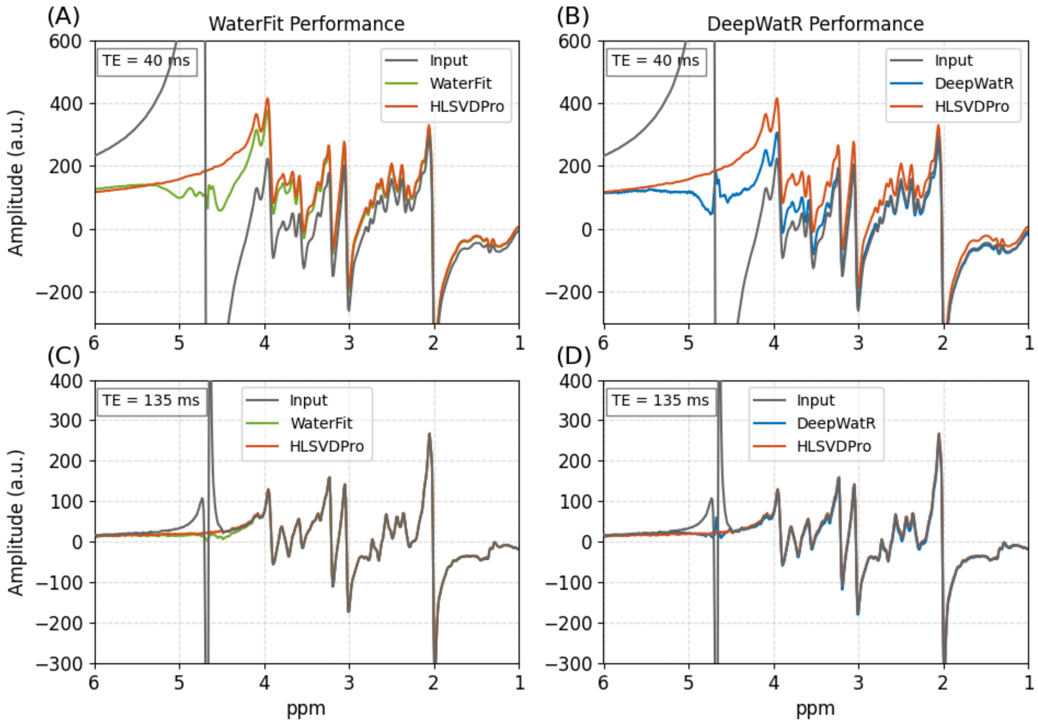

FIGURE S1 Water removal performance in dispersion mode. Average spectra with and without water residual compared against HLSVDPro and the original spectra. The first and second rows correspond to TE = 40 and TE = 135 ms, while the right and left columns correspond to WaterFit and DeepWatR, respectively.

Supporting Information 2

Figure S2 presents the histogram of the mean residual signal in the water range between 4.4 and 5.0 ppm for WaterFit, DeepWatR, and HLSVDPro on both real and imaginary parts in first and second row, respectively. The data used for this plot corresponds to the validation dataset of the main text, shown in Figure 3. Figure S2 A) and C) corresponds to an echo time of 40 ms, while B) and D) shows the cases for the long echo time of 135 ms.

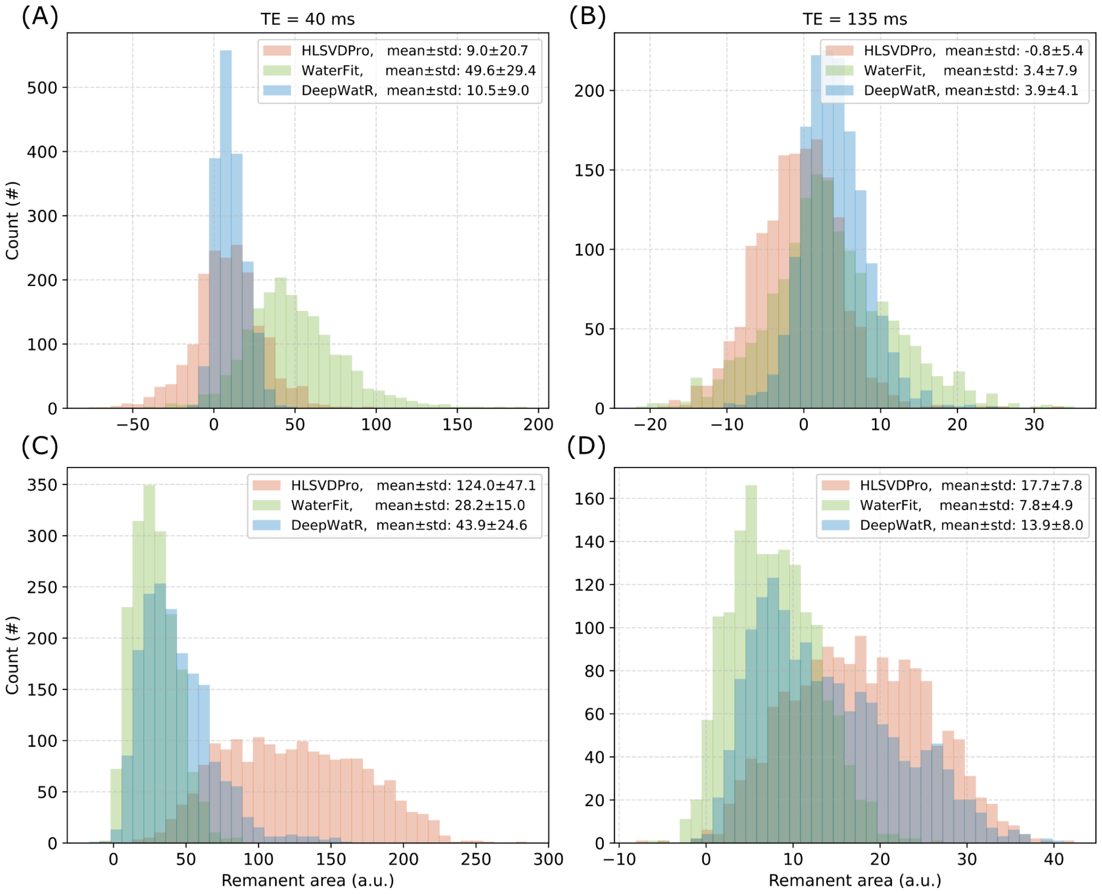

FIGURE S2 Histogram of the mean amplitude of residual water for both real (top row) and imaginary (bottom row) part after removal for each method used with TE = 135 (right column) and TE = 40 ms (left columns). The mean and standar deviations of each distribution is shown in each plot legend.

Supporting Information 3

Figure S3 shows the performance of each method while removing water in metabolites with a macromolecular baseline. WaterFit creates an extra baseline with a Lorentzian tail shape, which allows the correct fit of the whole spectrum using a baseline. In the DeepWatR case, the network creates a non-smooth Lorentzian tail, including peaks that overlap with the metabolites. This effect does not correctly fit the baseline and therefore results in a smaller metabolite quantification.

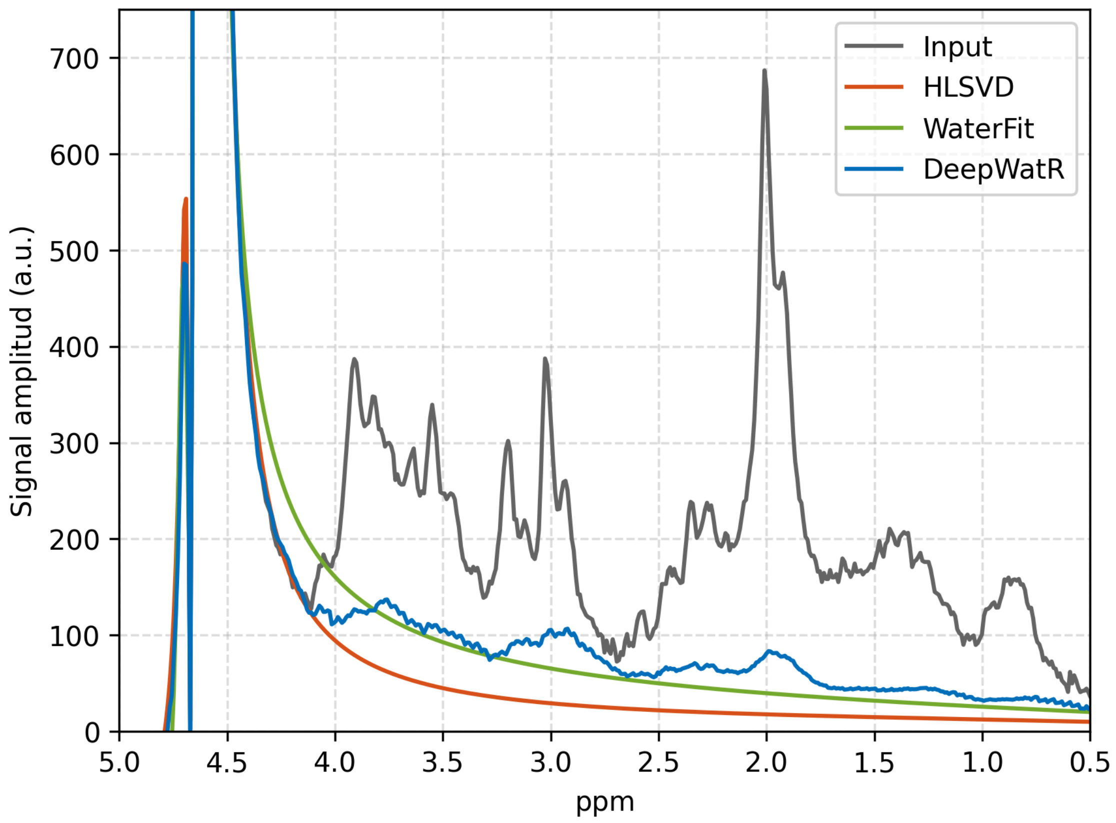

FIGURE S3 Performance of each residual water removal tool, Input corresponds to the original spectrum for a case with TE = 40 ms.

Supporting Information 4

To better understand the influence of water peak magnitude on the performance of WaterFit and DeepWatR, we present the differences between the two methods using HLSVDPro in the metabolite region. Figure S4 A) shows the average difference between the water peak obtained by WaterFit and HLSVDPro (y-axis), as a function of the max amplitude of the original water peak for both echo times. Figure S4 B) shows the same metric for DeepWatR, where better correlation with the peak amplitude is seen. Additionally, Figures S4 C) and D) show the same two metrics, but correlated with the area under the water peak, to account for water peak width.

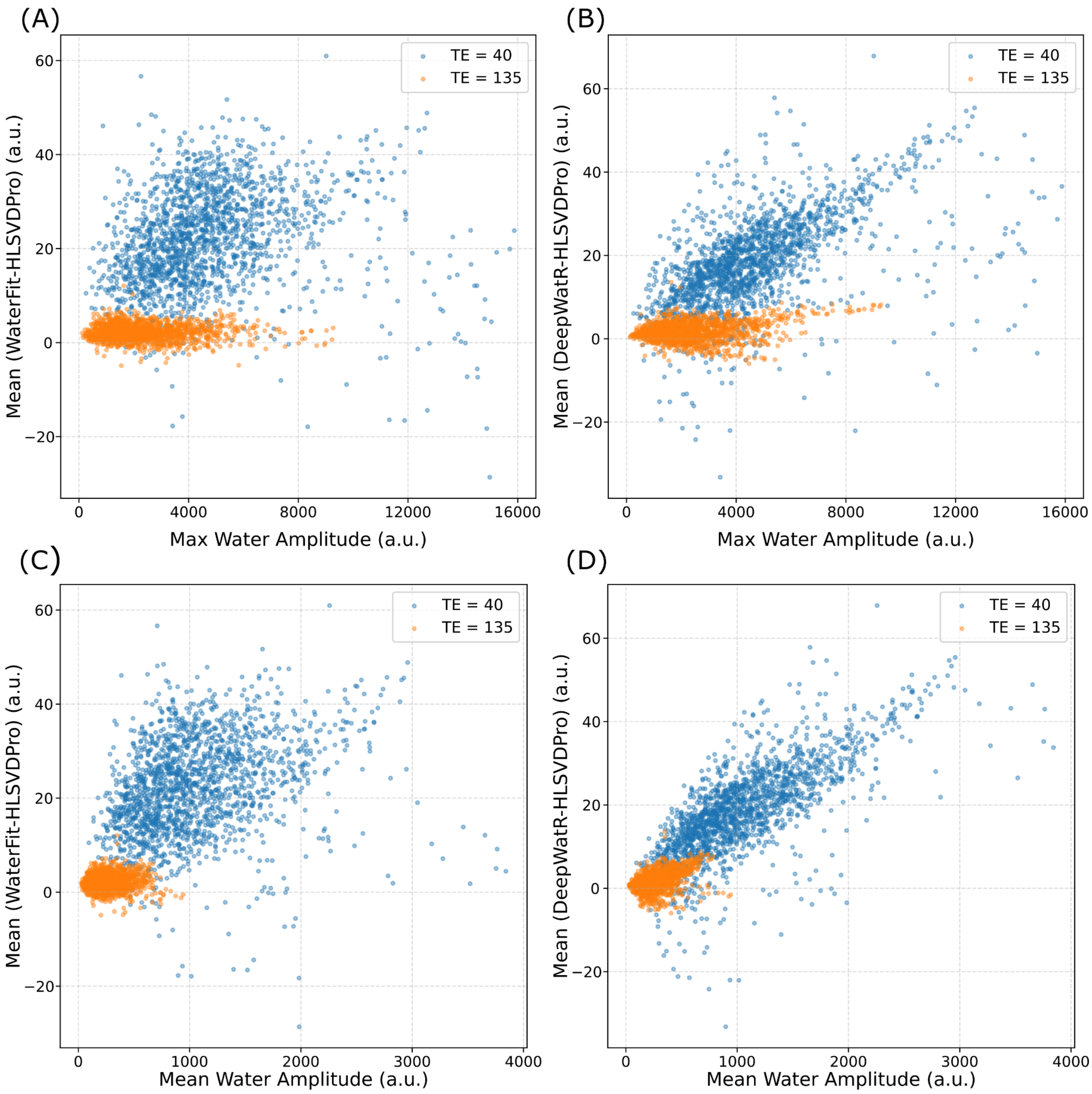

FIGURE S4 Comparison of water suppression performance between WaterFit and DeepWatR using HLSVDPro as a reference in the metabolite region. (A) Mean difference between WaterFit and HLSVDPro (y-axis) as a function of the maximum water peak amplitude, for TE = 40 ms (orange) and TE = 135 ms (blue). (B) Same as (A), but for DeepWatR instead of WaterFit. (C) The mean difference between WaterFit and HLSVDPro is plotted against the mean water amplitude, providing an estimate of the peak area. (D) Same as (C), but for DeepWatR.

Supporting Information 5:

To illustrate the performance of the three methods in cases of small and big water contributions, Figure S5 shows the original signal together with the water-removed signal obtained by HLSVDPro, WaterFit and DeepWatR. On the first row both sub-figures correspond to TE = 135 ms, where (A) corresponds to a case in the button 10% of water peak area, while (B) comes from the top 10% in water area. The second row is the same representation for the case of TE = 40 ms.

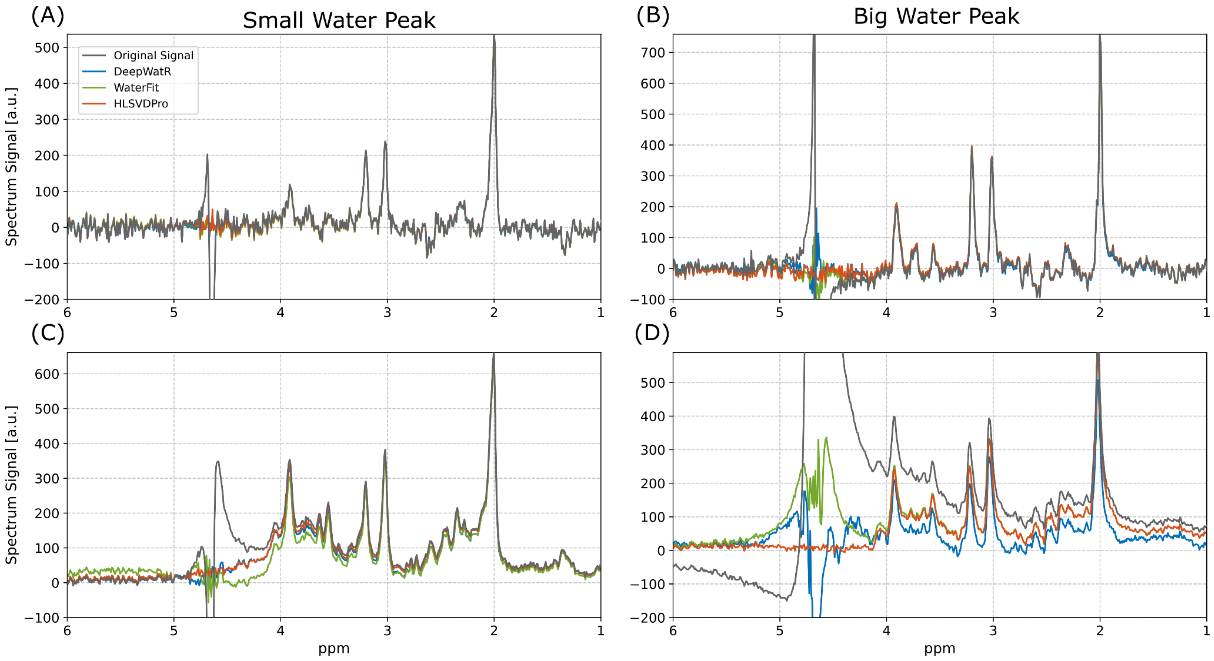

FIGURE S5 Example spectra showing water residual removal performance for cases with small (left) and large (right) water peaks. Top row (A–B) corresponds to TE = 135 ms; bottom row (C–D) to TE = 40 ms. Original spectra (black) are shown alongside results from DeepWatR (blue), WaterFit (green), and HLSVDPro (orange). Cases in (A) and (C) are from the bottom 10% of water peak area; (B) and (D) from the top 10%.

Supporting Information 6

Figure S6 presents a scatter plot for the main metabolites (Cho, Cre, NAA) amplitude with their corresponding r^2, and the first row corresponds to TE = 40 ms, while the second shows TE = 135 ms. In all cases, WaterFit removes water with less metabolite amplitude bias than DeepWatR. This effect is more pronounced in the case of TE = 40 ms, where the bias in DeepWatR is larger, as indicated by the r^2 value.

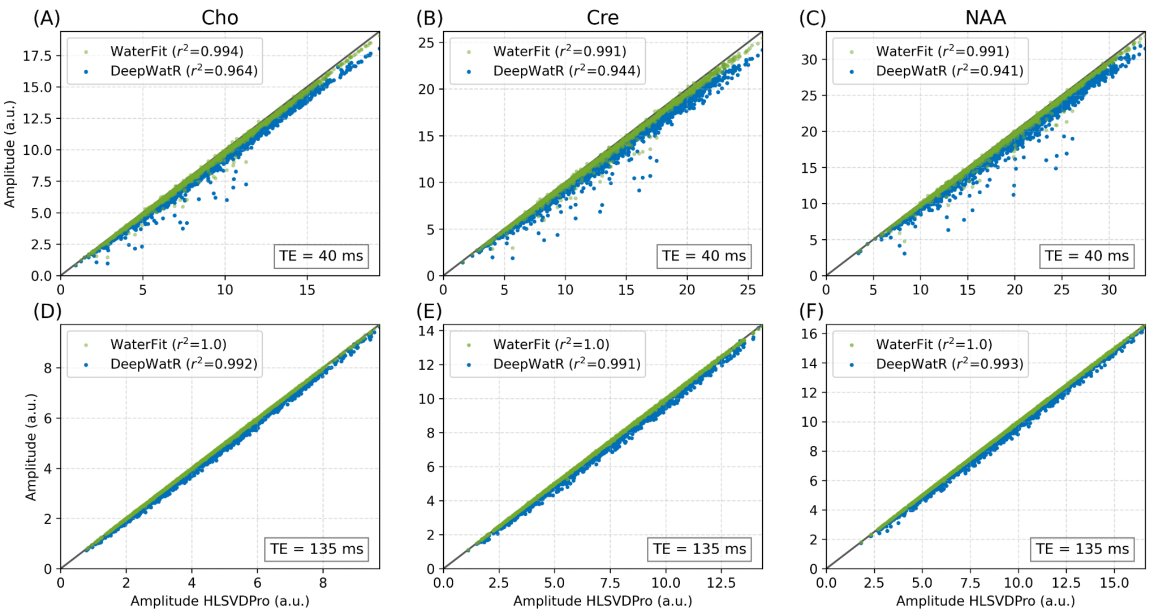

FIGURE S6 Scatter plot comparing the amplitude of main metabolites (Cho, Cre, NAA) between WaterFit and DeepWatR in the y-axis, and HSLVDPro in the x-axis for TE = 40 ms and TE = 135 ms in the first and second row, respectively.
